# Case Report: Monitoring neuromuscular fatigue through jump performance over two seasons in a cerebral palsy sprinter

**DOI:** 10.3389/fspor.2025.1558020

**Published:** 2025-03-07

**Authors:** Diego Antunes, Eduardo Marcel Fernandes Nascimento, Mateus Rossato, Edson Soares da Silva, Ricardo Dantas de Lucas, Gabriela Fischer

**Affiliations:** ^1^ Sports Center, Federal University of Santa Catarina, Florianópolis, Brazil; ^2^ Human Performance Laboratory, Federal University of Amazonas, Manaus, Brazil; ^3^ Inter-university Laboratory of Human Movement Biology, Université Jean Monnet Saint-Etienne, Saint-Etienne, France

**Keywords:** vertical jump, monitoring neuromuscular fatigue, cerebral palsy, case report, para athlete, para athletics

## Abstract

**Introduction:**

World Para Athletics classifies athletes with brain injury, cerebral palsy, and motor coordination impairments into Group Class 31 to 38. Para athletes who can run and jump but are affected by impairments such as athetosis, ataxia, and dystonia, which impact all four limbs and the trunk, are categorized as Class T36. Monitoring training load and performance is essential for guiding training programs and preventing injuries in this population. Vertical jumps are commonly used to assess neuromuscular parameters in athletes with cerebral palsy. In this study, we tracked performance changes and monitored vertical jump height and power over two competitive seasons in a sprinter with cerebral palsy.

**Case presentation/methods:**

The sprinter has had cerebral palsy since birth and is classified in the T36 class. Over two competitive seasons, his neuromuscular performance was monitored weekly using vertical jump tests, particularly Countermovement and Squat Jumps. His running performance was assessed through competition results. The parameters measured included Jump height and Peak power (W_PEAK_), which was calculated as the highest value from the curve obtained by multiplying the ground reaction force by the velocity during the concentric phase of the jump, normalized by body mass. Additionally, his official race times for the 100 m, 200 m, and 400 m events were recorded from January 2017 to October 2018.

**Results:**

The absolute and relative sprint performance values improved significantly between the first and the best official results: 100 m (from 15.05 s to 13.97 s = −7.1%), 200 m (from 31.30 s to 29.05 s = −7.1%); and 400 m (from 71.60 s to 66.24 s = −7.4%). The correlation between vertical jump parameters and sprint performance results was large to very large for the 100 m and 200 m events (*r* = 0.55–0.87).

**Discussion:**

The Para athlete demonstrated improved performance over two seasons and didn't sustain any injuries. These findings suggest that monitoring jump performance is a valuable and practical method for tracking training loads and predicting sprint performance. Further, longitudinal studies are needed to investigate the applicability of vertical jumps as a tool for coaches to monitor training load and performance across athletes with CP from various track classifications and event types.

## 1 Introduction

The Paralympic classification is designed to minimize the impact of disabilities on Para athletes’ performance and competition outcomes. It determines athletes’ eligibility and assigns them to Sports Classes based on the impact of their impairment on fundamental skills required for the Para sport (1). Para athletes, with brain injuries, cerebral palsy (CP), and motor coordination impairments are assigned to Group Class 31–38. Within this group, athletes who can run and jump using their lower limbs but are affected by impairments such as athetosis, ataxia, and dystonia, impacting all four limbs and the trunk, are classified as Class T36 (2). These athletes face performance limitations due to discoordination (3), biomechanical challenges (3), reduced muscle power, and lower-limb asymmetries (4–6). Between 1992 and 2012, the athletes with CP from class T37 obtained a remarkable 9% improvement in performance in the Paralympic games compared to less than 3% improvement among Olympic athletes over the same period (7). This highlights the need to investigate strategies for monitoring training and enhancing performance in this population.

Monitoring training load is essential for achieving optimal training adaptation. It provides individualized feedback and helps optimize training stimuli, making the process more efficient and tailored to the athlete (8). It also helps prevent inappropriate workloads that may lead to overuse injuries or overtraining syndrome (9). Assessing the daily promptitude of athletes is fundamental to ensure performance progress throughout the season and to identify dysfunctions related to exercise. Performance is characterized as the outcomes of a psycho-physiological complex interplay, encompassing physical and psychological well-being as well as competition readiness (10). In this context, evaluating neuromuscular function/performance appears to be an appropriate and practical method for tracking the accumulated fatigue process (11). In the training routines of sprinters, daily sessions often involve multiple muscle power stimuli, which makes it challenging to maintain an optimal balance of training load. Therefore, monitoring muscle power is a valuable approach for assessing key components of performance (12).

Vertical jump performance is a well-recognized method for assessing lower-limb muscle power (13, 14). The countermovement jump (CMJ) is a particularly effective tool for identifying neuromuscular fatigue in sprinters during and after exercise, as it provides insights into both mechanical and metabolic fatigue (15, 16). Vertical jump performance improvements correlate notably with increased sprint velocity in sprinters (17, 18). Jump tests demonstrate good reliability and validity in athletes with CP and are valuable tools for evaluating activity limitations in Para Athletics, particularly in classes T35–T38 (19). For football players with CP, jump tests such as CMJ and squat jump (SJ) have proven to be effective for assessing neuromuscular fatigue (20). Para Athletes from different classes T35–T38 exhibit varying performances, with lower performance levels often associated with more severe impairments (5). To the best of our knowledge, no previous study has conducted long-term monitoring of neuromuscular fatigue and performance in sprinter athletes with CP, particularly in those from classes with more severe impairments.

Studies that track trained CP athletes over extended periods are necessary to provide deeper insights into effective training prescriptions and the physiological effects of CP (21). This will advance the understanding of long-term training in individuals with CP. Given the unique characteristics of these athletes, investigating strategies for monitoring training and performance is crucial for injury prevention and for guiding tailored training programs. In this case report, we tracked changes in sprint performance and vertical jump height/power over two seasons (21 months) in a sprinter with CP, classified as T36 and corresponding to Gross Motor Function Classification System Level I.

## 2 Case description

The sprinter has had CP since birth (1991) due to oxygen deprivation during childbirth. According to CP clinical signs (athetoid/ataxic), he is classified in the T36 class. Throughout his life, he has been involved in sports. Before monitoring the training period, the athlete participated in an initiation process in Para athletics in different modalities (jump, throwing, and running) in 2015, and began training and competing in running events in 2016. During 2016 the athlete had several minor injuries such as abrasions and bruises due to falls and imbalances. Over two seasons (21 months), his performance was monitored using vertical jump tests, specifically CMJ and SJ, with weekly assessments conducted from January 2017 to October 2018. At the start of 2017, the sprinter was 25 years old. By the last Brazilian National Competition in 2018, he was 27 years old. The Para athlete is highly trained and competes at the national level (22). The athlete was informed of the risks and benefits of participation in this study and provided written informed consent. The study protocol was approved by the institutional ethics committee of the Federal University of Santa Catarina and conducted according to international standards (23). During the two seasons of training monitoring, the athlete attended eight official competitions, including two regional events, two qualifying meetings for nationals, and four national competitions. The local federation sanctioned the regional competitions, while both regional and national competitions were sanctioned by the Brazilian Paralympic Committee (CPB).

## 3 Methods

**Figure 1:**
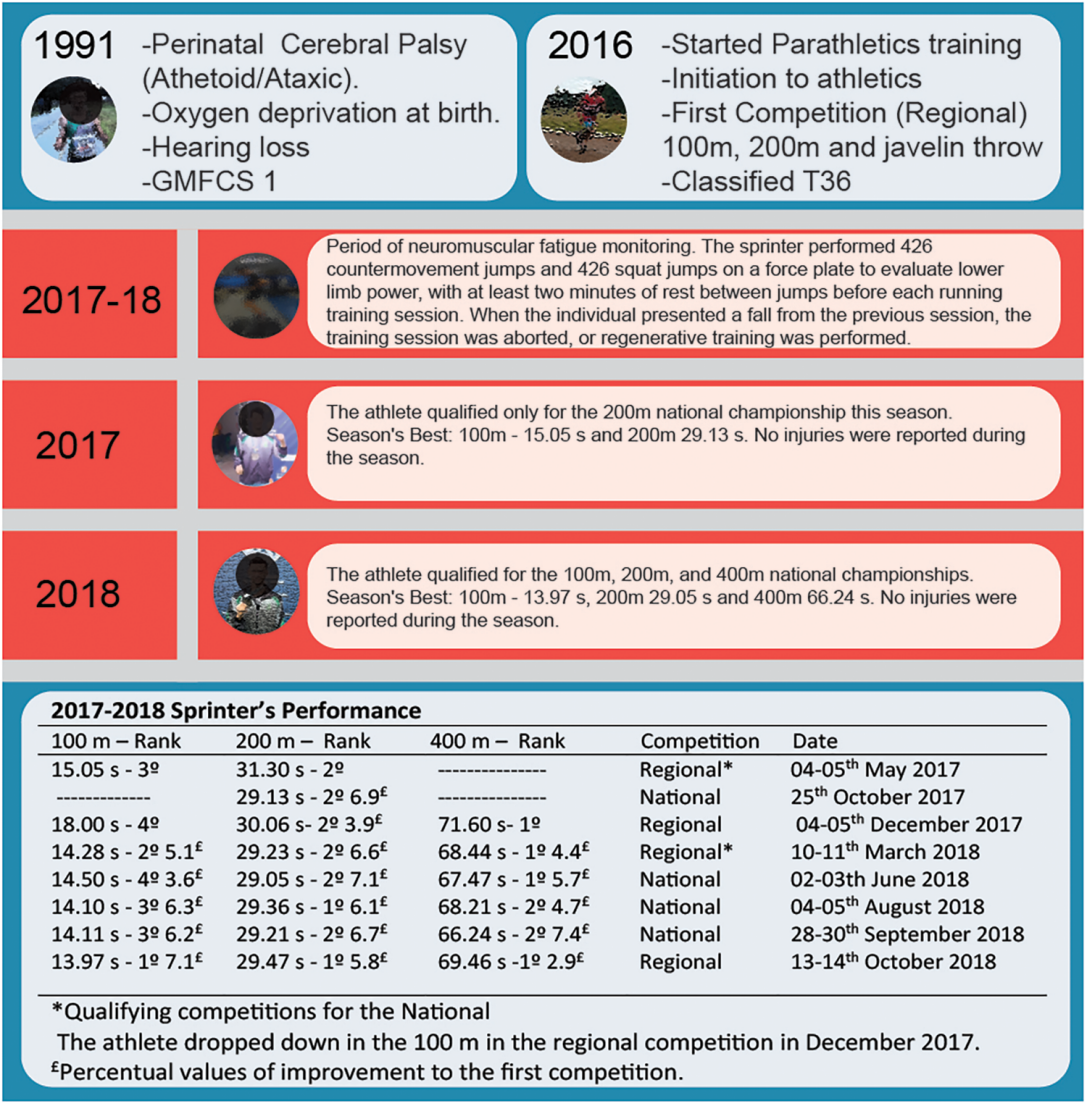
Showing case timeline with athlete's history. Gross Motor Function Classification System, GMFCS.

Before each training session, the athlete completed a 10 min cycling warm-up, followed by three CMJs and three SJs on a force plate (Quattro Jump, model 9290AD, Kistler, Winterthur, Switzerland) to assess lower-limb power. A minimum of two minutes of rest was allowed between jumps before each running training session. Data was acquired at a frequency of 500 Hz, with parameters calculated as follows: (a) Jump height: determined using the double integration method of the ground reaction force (GRF); and (b) Peak power (W_PEAK_): defined as the highest value from the curve obtained by multiplying the GRF by the velocity during the concentric phase of the jump, normalized by body mass. CMJ: The athlete began from a static standing position with hands on the hips. The jump was preceded by a counter-movement, lowering the center of gravity through knee flexion to approximately 90° as visually monitored by the examiner. During the jump, the trunk was maintained as vertically as possible, and the athlete was instructed to exert maximum effort to achieve the highest possible jump. SJ: The athlete initiated the jump from a static position, with the knees flexed to approximately 90°, the trunk maintained as vertically as possible, and the hands placed on the waist. The jump was executed without any countermovement, involving only the concentric action of the agonist muscles. The W_PEAK_ and jump height were monitored from January 2017 to October 2018. If the Para athlete's jump performance declined from the previous session by approximately 3%–5% in jump height, the training session was either canceled or replaced with light training consisting of coordination exercises. During this monitoring period, the Para athlete participated in eight official competitions, two regional events, two national qualifiers, and four national events championships, where the results were recorded (Figure 1).

## 4 Statistics

Values were expressed as means and standard deviations (±SD), with the highest values for each jump recorded. After ensuring Gaussian data distribution (normality by Kolmogorov–Smirnov test), a one-way ANOVA with Scheffe's *post hoc* test was applied to identify significant differences in monthly jump performance. To assess the variability in jump performance across seasons, we calculated and reported the mean, standard deviation, and coefficient of variation (CV) for the jump variables (CMJ height, SJ height, W_PEAK_CMJ, and W_PEAK_SJ) for both the 2017 and 2018 seasons. The CV was expressed as a percentage to facilitate the comparison of variability between variables and seasons. May 2017 was chosen as a reference for statistical comparison because it marked a peak performance phase before the competitive season. This time point was also selected based on data availability and consistency across sessions. Results were considered signiﬁcant at *p* < 0.05. Cohen's (24) (*d*) effect sizes were used, and the values were interpreted using the following scale: <0.20 (trivial), 0.2–0.6 (small), 0.6–1.2 (moderate), 1.2–2.0 (large), 2.0–2.4 (very large), and >4.0 (extremely large). To account for meaningful variations in vertical jump height during load monitoring, the Smallest Worthwhile Effect (SWE) was determined as 0.6 times the standard deviation (SD) of the jump height measurements. This threshold was chosen to ensure that only moderate or greater variations were considered significant, reducing the influence of trivial fluctuations (24). The Pearson correlation coefficient was used to examine the association between jump test performance (SJ and CMJ) and sprint performance across different competitions (100, 200, and 400 m) at national and international levels. Correlation values (r) were interpreted as follows: |<0.10|, trivial; |0.10–0.29|, small;|0.30–0.49|, moderate; |0.50–0.69|, large; |0.70–0.89|, very large; and |0.90–1.0|, almost perfect (25). Linear regression models were used to examine the relationship between jump performance and sprint times in the 100 m and 200 m races. Separate regression models were fitted for each race with race time as the dependent variable and jump variables as independent predictors. *p*-values were used to assess statistical significance, with a threshold of *p* < 0.05. All regression analyses were conducted using R version 4.4.2.

## 5 Results

**Figure 2:**
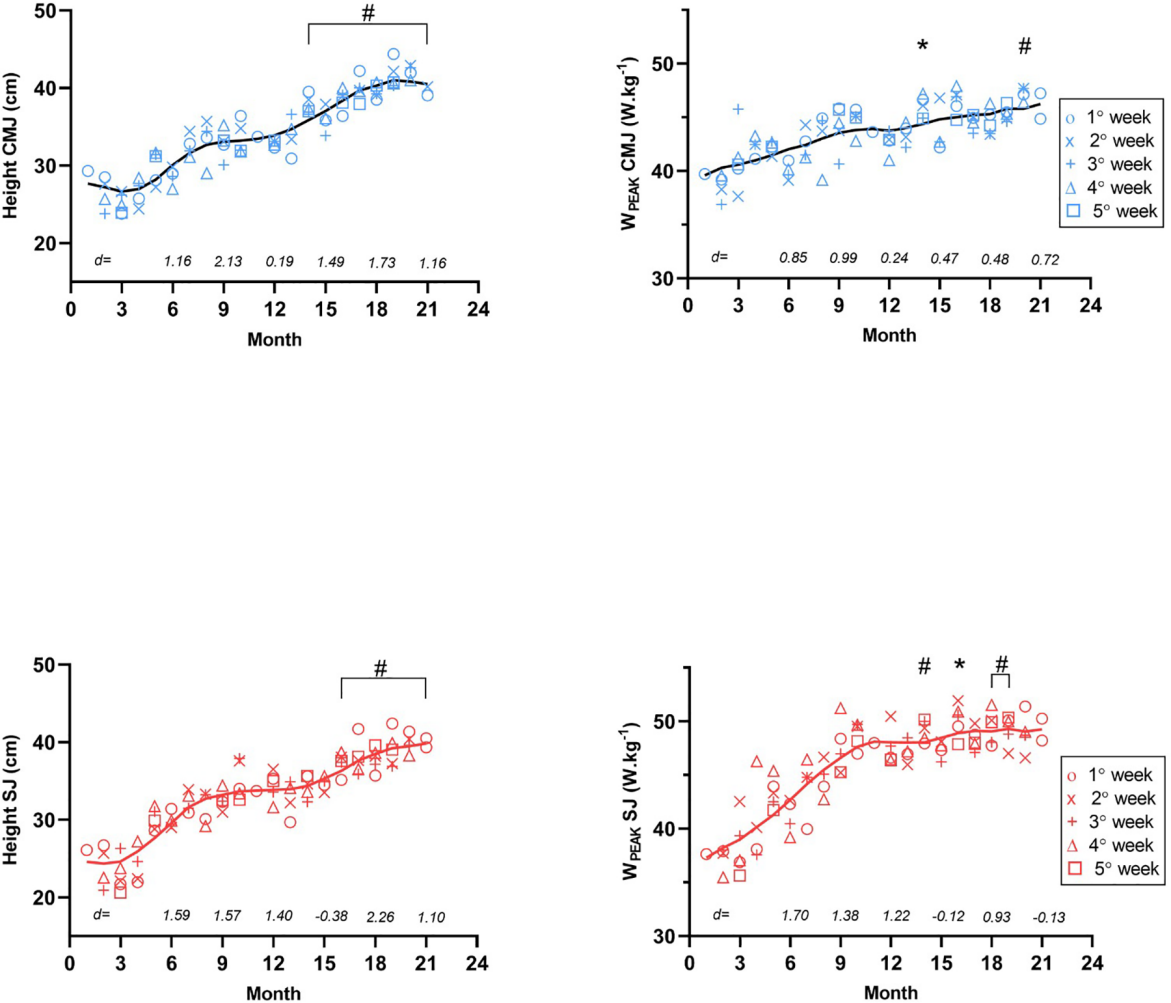
Vertical jumps tests verified week-to-week over the twenty-one months. *p < 0.05 and #*p* < 0.01 in relation to the first month of competition (fifth month—May 2017). The black line and red represent the data smoothing of jumps. *d* *=* represents the effect size value of a quarter compared to the previous quarter. The competitions took place in months 5(1° week), 10 (4° week), 11, 14 (1 ° week), 17(1° week), 19(1° week), 20(4° week) and 21(2° week). During the competition week in the 11th month, the jump was not recorded due to travel logistics.

**Figure 3:**
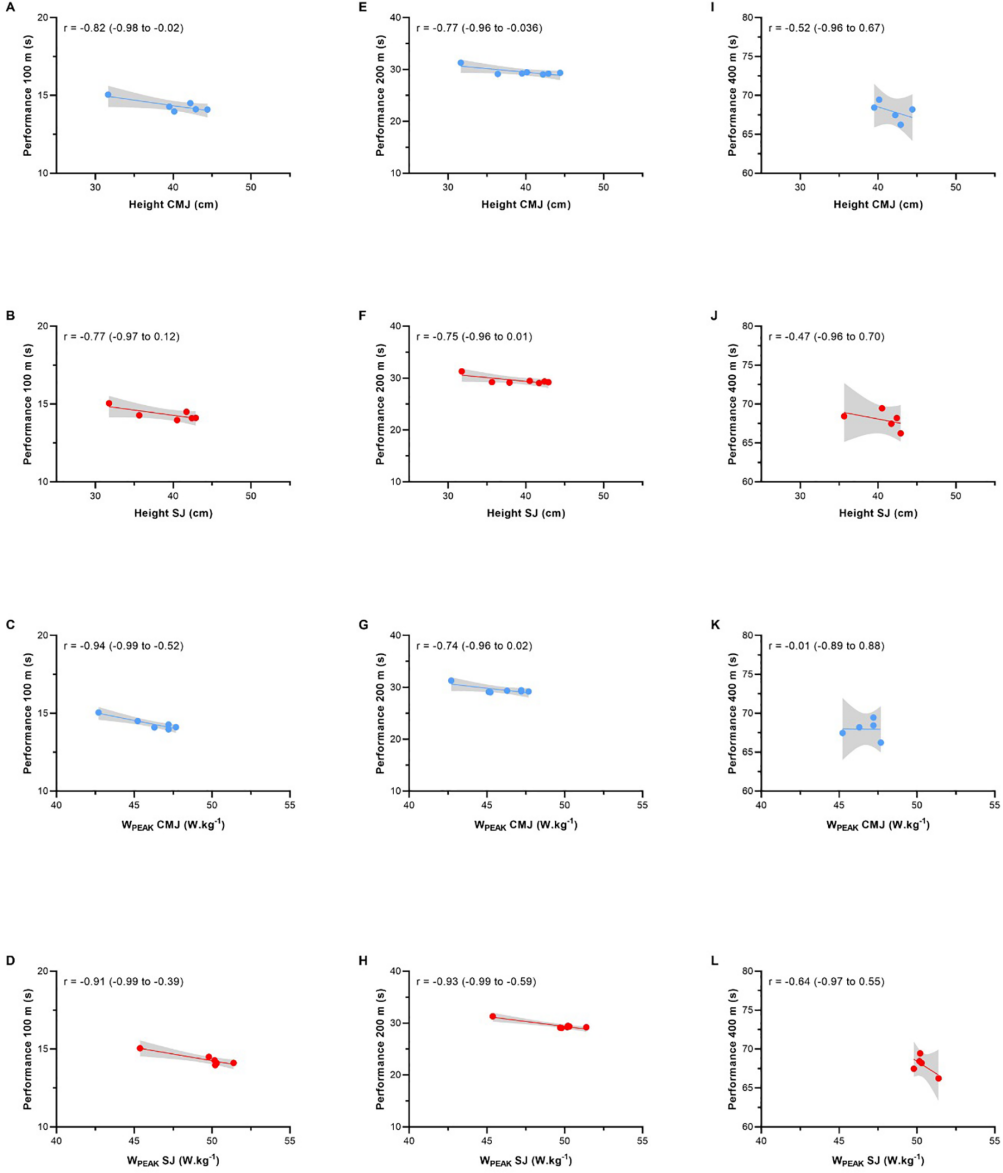
Relationship between vertical jumps tests and performance in competitions (100 m, 200 m and 400 m). **(A)** 100 m vs. height CMJ (cm), **(B)** 100 m vs. height SJ (cm), **(C)** 100 m vs. W_PEAK_ CMJ, **(D)** 100 m vs. W_PEAK_ SJ, **(E)** 200 m vs. height CMJ (cm), **(F)** 200 m vs. height SJ (cm), **(G)** 200 m vs. W_PEAK_ CMJ, **(H)** 200 m vs. W_PEAK_ SJ, **(I)** 400 m vs. height CMJ (cm), **(J)** 400 m vs. height SJ (cm), **(K)** 400 m vs. W_PEAK_ CMJ and **(L)** 400 m vs. W_PEAK_ SJ. CMJ, countermovement jump; SJ, squat jump; W_PEAK_, peak power.

No significant differences in body mass were observed across the seasons (*p* > 0.05). In 2017, CMJ height averaged 29.6 cm (SD = 3.61 cm, CV = 12.2%), increasing to 38.5 cm (SD = 3.18 cm, CV = 8.27%) in 2018. Similarly, SJ height had a mean of 28.7 cm (SD = 4.69 cm, CV = 16.3%) in 2017 and 36.9 cm (SD = 2.74 cm, CV = 7.42%) in 2018. Regarding peak power, CMJ showed an average of 42.0 W.kg^−1^ (SD = 2.68 W.kg^−1^, CV = 6.39%) in 2017 and 45.1 W.kg^−1^ (SD = 1.97 W.kg^−1^, CV = 4.37%) in 2018, while SJ peak power increased from 42.4 W.kg^−1^ (SD = 4.68 W.kg^−1^, CV = 11.0%) in 2017 to 48.7 W.kg^−1^ (SD = 1.99 W.kg^−1^, CV = 4.09%) in 2018. Significant differences in jump performance were found between the first month of competition (May 2017) and the fourth month of competition (March 2018) based on weekly measurements over the 21 months (Figure 2). The effect size values of a quarter compared to the previous quarters are in detail in Figure 2. The jump height SWE range among sessions was approximately 3%–5%. Absolute and relative sprint performance showed improvements between the first and best official results: 100 m (from 15.05 s to 13.97 s = −7.1%), 200 m (from 31.30 s to 29.05 s = −7.1%); and 400 m (from 71.60 s to 66.24 s = −7.4%). The correlations between vertical jump parameters and sprint performance were large to very large for the 100 m and 200 m events (*r* = 0.74–0.94), but trivial to moderate for the 400 m event (*r* = 0.01–0.51) (Figure 3). Linear regression with jump variables demonstrated a good model fit for predicting race times in both the 100 m and 200 m (*R*^2^: 89.41% and 90.21%, respectively). However, none of the jump variables (height or power) showed a significant association with the 100 m race time. In the 200 m, W_peak_SJ showed a marginally significant association with race performance (*p*-value 0.05150), while other variables did not show a significant relationship.

## 6 Discussion

The primary finding of this case report indicates that monitoring vertical jump performance and estimating weekly variability over a 21-month period in a sprinter with CP is an effective approach for tracking training load and sprint performance throughout the competitive season. Practical and effective methods, such as CMJ and SJ evaluations, provide coaches with straightforward insights into critical performance variables. Competition results in the 100 m, 200 m, and 400 m events demonstrated consistent improvement from the first competition, with performance gains ranging from 2.9% to 7.4% (Figure 1). The lower coefficient of variation observed in 2018 across all jump variables suggests greater consistency in performance compared to 2017. Furthermore, jump-derived variables, including jump height and peak power (W_PEAK_), strongly predict improvements in sprint performance in the 100 m and 200 m events (Figure 3). However, to explore causality, we conducted regression analyses. While the models showed predictive power (*R*^2^ = 89.41% for the 100 m and 90.21% for the 200 m), the limited sample size and focus on a single athlete prevent causal conclusions. Despite the different characteristics between visual impairment sprinters and CP sprinters, a previous study suggests that jump variables are reliable predictors of sprint performances in paralympic events (12). Notably, the strongest correlations were observed with W_PEAK_ normalized by the body mass. These results suggest that, possibly, the ground contact time and the force generated during this phase directly influence running performance, particularly in individuals with brain injuries who experience neuromuscular activation impairments. A study conducted by Bezodis et al., (26) compared a sprinter with cerebral palsy (one elite T36 class) to non-disabled sprinters (16 well-trained), analyzing kinematics during 10-meter sprints. The results indicated that vertical force did not show significant differences between the T36 sprinter and non-disabled sprinters. However, horizontal force exhibited notable discrepancies, particularly in the final two-thirds of the test. Nevertheless, as that study examined only a single athlete with cerebral palsy, conclusions regarding the relative influence of horizontal and vertical forces on the performance of sprinters with brain injuries or in the T36 class remain limited. Practical methods like the CMJ and SJ tests used to monitor neuromuscular fatigue, provide accessible performance metrics while aligning with competition-specific movements. These methods are crucial for improving performance and preventing injuries, particularly in athletes with brain injuries who are more vulnerable to upper and lower limb injuries (27).

In this case report, vertical jump performance was used to monitor the training load and training prescriptions. If the Para athlete's jump height decreased by approximately 3%–5% compared to the previous session, the training session was either canceled or substituted with light coordination exercises. Notably, the athlete remained injury-free throughout the study period. Vertical jumps, particularly CMJ, have been widely used to detect neuromuscular fatigue in athletes without disabilities (16, 28). The utility of the CMJ test is supported by a strong correlation between CMJ height loss and metabolic responses during exhaustive training sessions in sprinters (15, 29). In an investigation, vertical jumps demonstrated good reliability and validity in CP football players. The authors suggest that vertical jumps may apply to the evaluation of Para Athletics athletes in classes T35–T38 (19). In this case, despite significant levels of discoordination, asymmetries, and reduced lower-limb strength characteristic of an athlete from class T36 compared to higher classifications (T37–T38) (4), the Para athlete presented a low CV over the months, with jump performance remaining consistent within approximately 1 cm. Additionally, in CP footballers, vertical jump height has shown significant decreases after matches and is associated with internal load measures (20). Individuals with brain injuries often report fatigue accompanied by pain (30). Therefore, it is crucial to differentiate between physiological fatigue caused by training or over-reaching, and pathological fatigue linked to a disease or brain injury (31), as well as to consider the influence of the impairment level.

Sprinters with brain injuries in classes T37–T38 and athletes without disabilities exhibit similar levels of fatigue following exhaustive exercises, suggesting that high-level training over many years may help mitigate the effects of impairments (32–34). However, there is limited information on fatigue in sprinters with great impairments, such as those in classes T35 and T36. Sprinters with CP and more severe impairments demonstrated lower jumping performance, reduced strength, and greater asymmetries between their lower limbs (4). A deeper understanding of the rate of fatigue development and recovery in individuals with CP is crucial, particularly in determining whether the severity of fatigue correlated with the level of impairment (30).

To our knowledge, this is the first longitudinal study to monitor training load in a sprinter with a brain injury using vertical jump tests. Notably, the athlete did not develop any injuries over the course of two competitive seasons, a critical factor in performance improvement, particularly for individuals with brain injuries who experience movement dysfunctions associated with the pyramidal or extrapyramidal tracts (31). Future studies should incorporate analyses of variables derived from jump tests alongside kinetic data, such as biomechanical factors, flight time, ground contact time, step frequency, and step length, particularly on the non-involved or less involved side, which are closely linked to performance in athletes with brain injuries (3). Additionally, running coordination factors should be further explored (5). This case report has considerable limitations. First, due to the unique characteristics of athletes across the T35, T36, T37, and T38 sports classes, and even within the T36 class itself, the findings of this report cannot be generalized to all Para athletes. Second, other factors that may have contributed to improvement in performance and injury prevention over the two years of load monitoring were not assessed. These include variables such as motivation, prior athletic experience, familiarity with the testing protocols, strength training, nutrition, and weather conditions. It is important to consider a bias possibility in data collection. Third, the small sample size is based on data from a single athlete and a limited number of competitions. This restricts the generalizability of the findings and prevents us from drawing causal conclusions. Future studies with larger sample sizes and longitudinal or experimental designs are needed to better understand the relationship between jump performance and sprint times. Despite these limitations in fully capturing the reality of all athletes, the present study monitored a well-trained Para athlete over two competitive seasons, using vertical jump assessments and data from eight official competitions. The Para athlete, a National Champion, was also licensed to compete at the international level. In conclusion, the vertical jump performances correlate with sprint performances (100 m and 200 m) in a class T36 athlete with CP over two seasons. However, further longitudinal studies are needed to investigate the applicability of vertical jumps as a tool for coaches to monitor training load and performance across athletes with CP from various track classifications and event types.

## Data Availability

The raw data supporting the conclusions of this article will be made available by the authors, without undue reservation.
